# Awareness of prostate cancer and its associated factors among men 40 years of age and older in Mizan Aman town, Bench Sheko zone, Southern Nations, Nationalities, and Peoples’ Region, South West Ethiopia, 2019

**DOI:** 10.3389/fonc.2022.976810

**Published:** 2022-10-24

**Authors:** Ashenafi Assefa, Gugsa Nemera Germossa, Mengistu Ayenew, Gadisa Bekele Bedada

**Affiliations:** ^1^ Department of Nursing, College of Health Science, Mizan Tepi University, Mizan, Ethiopia; ^2^ Department of Nursing, Institute of Health, Jimma University, Jimma, Ethiopia; ^3^ Department of Public Health, College of Medicine and Health Science, Mizan Tepi University, Mizan Aman, Ethiopia

**Keywords:** prostate cancer, awareness, men, Mizan Aman, Bench Sheko

## Abstract

**Background:**

Prostate cancer is a common cause of morbidity and mortality among men aged 40 years and older. Evidence has shown that awareness of prostate cancer plays a greater role in the early detection of prostate cancer. However, there is a paucity of information regarding prostate cancer awareness levels in Ethiopia in general and in Mizan Aman town in particular.

**Objectives:**

To assess the awareness of prostate cancer and its associated factors among men aged 40 years and older in Mizan Aman, Bench Sheko zone, Southern Nations, Nationalities, and Peoples’ Region, South West Ethiopia, 2019.

**Methods:**

A community-based cross-sectional study was conducted from 1 to 30 April 2021 in Mizan Aman town. A total of 322 study subjects were selected from a total population of 1,242 in Mizan Aman town by using the simple random sampling method. Data were collected through a face-to-face interview using a structured questionnaire. Data were entered into EpiData version 3.1 and analyzed by Statistical Package for Social Science version 20. Descriptive statistics were used to summarize sociodemographic characteristics and personal history. Bivariate and multivariate regression analyses were used to explore further variables that were associated with the level of awareness. Significant associations were declared at a *p*-value of <0.05. The results were presented in text, tables, and charts.

**Results:**

The magnitude of prostate cancer awareness was 64%. Participants who were between 40 and 55 years of age (adjusted odd ratio = 6.16, 95% confidence interval = 2.62–14.47), who were government employees (adjusted odd ratio = 4.684, 95% confidence interval = 1.56–13.97), and whose monthly income level is greater than 5,000 birr (adjusted odd ratio = 12.45, 95% confidence interval = 3.2–47.77) were significantly associated with the level of awareness.

**Conclusion and recommendation:**

This study revealed that more than half of the men residing in Mizan Aman town had a high level of prostate cancer awareness. First-category age, better economic status, and employment were significantly associated with awareness of prostate cancer. This indicates the need for a collective effort to enhance the awareness of men regarding prostate cancer.

## Introduction

Prostate cancer is ranked as the second most frequent and the fifth-leading cause of cancer death in the male population. Globally, in 2018 alone, an estimated 1.3 million new cases and 359,000 prostate cancer-associated deaths were recorded (1). In Africa, the pooled estimated prostate cancer incidence rate is 22.0/100,000 population with a median incidence rate of 19.5/100,000 population (2). Cancer of the prostate is an important public health problem in Africa (3). Also, according to a systematic review and meta-analysis conducted on prostate cancer, the incidence of prostate cancer in Africa is high. According to the GLOBOCAN 2020 report, there are 2,670 cases of prostate cancer in Ethiopia (4). In a cross-sectional study done in Addis Ababa, Black Lion Hospital, prostate cancer accounts for 2.6% in Addis Ababa (5). Currently, the burden of prostate cancer in Ethiopia is high. According to the WHO data published in 2020, prostate cancer deaths in Ethiopia reached 1,244 or 0.22% of the total deaths (6).

Prostate cancer can be diagnosed through a collection of tests and procedures such as a digital rectal examination (DRE), prostate-specific antigen (PSA) blood test, and prostate biopsy (7).

The risk factors contributing to prostate cancer were being African-American and Jamaican men, family history, and diets high in red meats and fatty foods and low in fruits and vegetables (8).

The prognosis of a patient with prostate cancer depends on the time of detection; that is, the earlier the detection of prostate cancer, the better the prognosis (9). Prostate cancer diagnosis, prevention, and treatment in recent decades have been heavily influenced by awareness and the intention to seek care for it. Men who were aware of the DRE/PSA test were more likely to have been screened compared to men who were not aware of it (9).

A good awareness or understanding of diseases is generally associated with a better healthcare-seeking attitude and behavior (10). According to a study conducted in Uganda in 2014 and 2013, low awareness related to late diagnosis of prostate cancer is a major reported problem (11, 12). A good level of awareness of prostate cancer is associated with a decrement in morbidity and mortality associated with the disease (13). However, different studies around the world report that the awareness level of prostate cancer is low.

An American study shows that there is still a low level of awareness of prostate cancer (14). Also, a cross-sectional study conducted on prostate cancer awareness and screening among men in a rural community in Kenya showed that 57.3% had a low level of awareness (15). Also, a study conducted in Ethiopia reported a low level of knowledge of prostate cancer, which is 41.2% (16). A low level of awareness of prostate cancer was associated with ethnicity (11), income level (14), occupation (15), religion (14, 17), age (18), educational status (14, 18), family history of prostate cancer (17), smoking (18), and marital status (19). Even though information about men’s level of awareness about prostate cancer is necessary for early screening and detection of the disease, there is a paucity of information regarding prostate cancer awareness levels in Ethiopia in general and in Mizan Aman town in particular. Thus, the study aimed to assess the awareness level of prostate cancer and its associated factors in Mizan Aman town.

## Methods and materials

### Study area and period

A community-based descriptive cross-sectional design was conducted from 1 to 30 April 2019 on male residents of Mizan Aman town. Mizan Aman, the capital town of Bench Sheko zone, is located 561 km away from Addis Ababa, the capital city of Ethiopia. Bench Sheko zone is part of the Southern Nations, Nationalities, and Peoples’ Region (SNNPR). The town has a total population of 72,860, of which 34,765 are men and 38,095 are women. The total population of men aged 40 years and older is 1,242. Men aged 40 and older who had lived in Mizan Aman town for more than 6 months were included in the study, while men who were too sick to respond and had known hearing impairment and mental illness were excluded.

### Sample size determination and sampling technique

**Figure 1:**
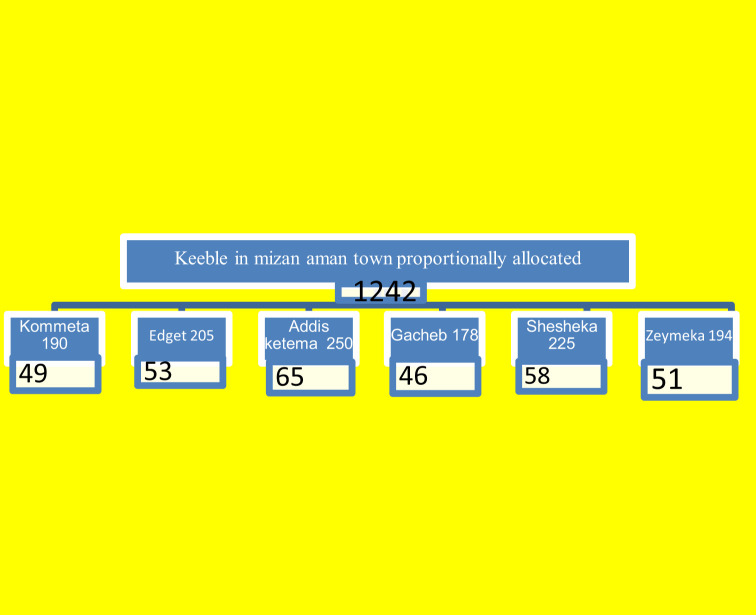
Schematic presentation of the sampling procedure in the study of awareness of prostate cancer in men 40 years of age and older in Mizan Aman.

The sample size was computed based on the single population proportion formula and using the prevalence of awareness of 50% because no study was done in Ethiopia. The *Z*-value was 1.96 at a 95% confidence interval and the margin of error was 5%. Accordingly, the calculated sample size was 384. Hence, the study population is less than 10,000, so we used a population correction formula. Formulas were used and the final sample size with a 10% non-response rate was 322 after correction. Of the total of six kabala, a man 40 years of age or older was found in 1242 in Mizan Aman town, of the total population. A simple random sampling method was used to select the men in Mizan Aman town for the study until a target sample size of 322 was achieved. A simple random sampling technique was used to get study subjects until a sample size of 322. (**Figure 1**; **Table 3**).

#### Variables of the study

The dependent variable is awareness of prostate cancer. The independent variables were as follows: age, education levels, occupation, religion, economic status, ethnicity, marital status, family history, income levels, smoking, and alcohol.

#### Measurement

*Awareness* refers to being aware of a situation or fact. In this study, it was measured by 24 items of awareness questions (20). Respondents who were able to answer greater than or equal to 50% of the total awareness questions were considered to have a *high level of awareness* (21). Those respondents who were able to answer less than 50% of the total awareness questions were considered to have a *low level of awareness* (21).

### Data collection tool and procedure

A data collection instrument was adapted from other similar studies done in other countries (21, 22). The tools have 24 items and are arranged into the following four parts: part I, sample characteristics (7 items); part II, history (2 items); part III, risky lifestyle (3 items); and part IV, prostate cancer awareness (12 items). Three B.Sc. nurses collected the data, and one M.Sc. nurse supervises the overall activity of data collection.

### Data quality control

The questionnaire was translated from English to the local languages “Benchagna” and “Amharic” by two language experts and then re-translated back to English by another language expert to check for consistency. A pretest was conducted on 5% of the sample (16 individuals) in neighboring Temenjeyaje town, which is 18 km from the study area, 1 week before the actual data collection to identify any ambiguity and consistency. The validity of the questionnaires was checked by two experts. The reliability of the items was determined through Cronbach’s alpha (0.84) for the awareness questions. The data collection process was supervised by the supervisor, who checked the completeness of the collected data daily. Training regarding the objective of the study, the maintenance of ethical standards, the methods of data collection, and how to recruit the participants was provided by the principal investigator to the data collectors and supervisors. The collected data were checked for its completeness at the end of the interview and at the end of the day by the supervisor. Data coding and cleaning were performed by cross-checking the printout data for obvious errors. Missing values and outliers were checked before analysis by running a descriptive analysis.

### Data processing and analysis

The completed questionnaire was checked for completeness and consistency and entered into EpiData statistical software version 3.1 and exported to SPSS version 21 for further analysis. Descriptive statistical analysis such as simple frequencies, measures of central tendency, and measures of variability was used to describe the characteristics of the participants including sociodemographic characteristics and the risk factors of prostate cancer.

Then, information was presented using a frequency distribution table and chart. For the analysis of the outcome variable, high-level awareness was recorded as 1, and low levels of awareness were recorded as 0. Bivariate analysis was used to assess the association between each independent variable and dependent variable using binary logistic regression. All variables with a *p*-value ≤0.25 were taken into the multivariable model to control for all possible confounders. Before we classified the level of awareness into high and low, we used the multicollinearity test for each unit of awareness independently, and then we classified the level of awareness using the Hosmer–Lemeshow test for independence. The odds ratio was used as the primary measure of the strength and direction of the relationship between the independent variables. Odds ratio along with 95% CI was estimated to identify factors associated with the level of awareness using multivariate analysis in the binary logistic regression. The level of statistical significance was declared at a *p*-value <0.05.

## Results

### Sociodemographic characteristics

A total of 322 respondents participated in the study with a 100% response rate. The respondents have different sociodemographic characteristics. The ages of the respondents ranged between 40 and 97 years with a median age of 49 years, and the majority (63%) of them were in the age category between 40 and 55 years. The majority (70.2%) were married, 39.4% completed tertiary education, and more than half (52.2%) were government employees. Regarding religion, the majority of the respondents were Orthodox and Protestant [150 (46.6%) and 130 (40.4%), respectively], and half of them were Bench by ethnicity. Concerning their socioeconomic status, the mean monthly income of the study participants was 3,470 birr (for details, see **Table 1**).

**Table 1:** Sociodemographic characteristics of men 40 years of age and older in Mizan Aman town, Bench Sheko zone in SNNPR, South West Ethiopia, 2019.

Variable	Categories	Frequency	Percent
Age (years)	40–55	203	63
	56–65	57	17.7
	>65	62	19.3
Marital status	Single	23	7.1
	Married	226	70.2
	Widowed	33	10.2
	Divorced	40	12.4
Educational status	Illiterate	26	8.1
	Read and write only	49	15.2
	Primary	55	17.1
	Secondary	65	20.2
	Tertiary	127	39.4
Occupation	Unemployed	30	9.3
	Daily worker	39	12.1
	Merchant	65	20.2
	Government employee	168	52.2
	Other	20	6.1
Religion	Orthodox	150	46.6
	Muslim	18	5.6
	Protestant	130	40.4
	Hawaryat	24	7.5
Ethnicity	Bench	161	50
	Keffa	71	22
	Amhara	32	9.9
	Oromo	18	5.6
	Sheko	40	12.4
Income in ETB	<2,000	75	23.3
	2,000–5,000	219	68
	>5,000	28	8.7

### Histor and risk factors

The study revealed that the majority of the study respondents (302, 93.3%) had no family history of prostate cancer and that 292 (90.7%) had no regular physical exercise (**Table 2**).

**Table 2:** History and risk factors of prostate cancer in men 40 years of age and older in Mizan Aman town, Bench Sheko zone in SNNPR, South West Ethiopia, 2019.

Variable	Frequency	Percent
Relatives with prostate cancer		
Yes	20	6.2
No	302	93.3
Family members with prostate cancer		
Father	4	1.2
Brother	7	2.2
Others^a^	9	2.2
Regular physical exercise		
Yes	30	9.3
No	292	90.7
Drink alcohol regularly		
Yes	30	9.3
No	292	90.7
Smoke regularly		
Yes	49	15.2
No	273	84.8

a Others: sister, uncle, or aunt with prostate cancer awareness.

### Prostate cancer awareness

Regarding awareness of prostate cancer, above half of study respondents said prostate cancer is malignant. 206(64%) and 219(68%) respondents said prostate cancer increases with age and screening is important for prostate cancer respectively. 290(90.1) of them responded as drinking, (**Table 3**).

**Table 3:** Awareness questions about prostate cancer in men 40 years of age and older in Mizan Aman town, Bench Sheko zone in SNNPR, South West Ethiopia, 2019.

Awareness questions	Yes, *n* (%)	No, *n* (%)
Prostate cancer is a common malignancy.	198 (61.5)	124 (38.5)
The risk of prostate cancer increases with age.	206 (64)	116 (36)
Is it important to be screened for prostate cancer?	219 (68)	103 (32)
DRE and PSA tests are used for prostate cancer screening or diagnosis.	14 (4.3)	308 (95.7)
Difficulty in micturition is a complaint of prostate cancer.	190 (59)	132 (41)
Prostate cancer is curable.	220 (68.3)	102 (31.7)
Prostate cancer is a preventable disease.	213 (66.1)	109 (33.9)
Physically inactive people are at risk of prostate cancer.	204 (63.4)	118 (36.6)
Prostate cancer can cause death.	212 (65.8)	110 (34.2)
Drinking alcohol is a risk factor for prostate cancer.	32 (9.9)	290 (90.1)
A high-fat diet is a risk factor for prostate cancer.	43 (13.4)	279 (86.6)
Smoking is a risk factor for prostate cancer.	36 (11.2)	289 (88.8)

**Figure 2:**
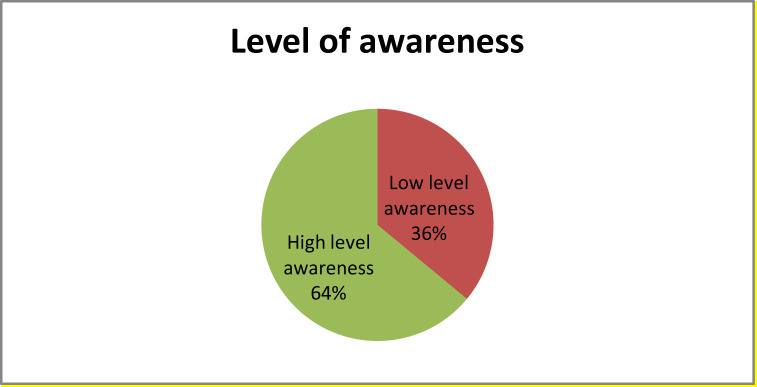
Level of awareness of prostate cancer in men 40 years of age and older in Mizan Aman town, Bench Sheko zone in SNNPR, South West Ethiopia, 2019.

This study revealed that 64% of the study respondents had a high level of prostate cancer awareness (**Figure 2**).

### Factors associated with prostate cancer awareness

In multivariate logistic regression analysis age, occupation and economic status were showed a statistically significant association with the level of awareness of prostate cancer. (**Table 4**)

**Table 4:** Bivariate and multivariate logistic regression analyses of men 40 years of age and older in Mizan Aman town, Bench Sheko zone in SNNPR, South West Ethiopia, 2019.

Variables	Level of awarness		COR(95% CI)	P*value	AOR( 95% CI)	P*value
	low level of awarnes in Number, %	high level of awarnes in Number, %				
**Age**						
40-55	49 (24.1)	154 (75.9)	4.65 (2.55-8.48)	0.000	6.2 (2.91-13.21)	<0.001
56-65	30 (52.6)	27 (47.4)	1.33 (0.64-2.75)	0.44	1.65 (0.69-3.9)	0.26
>65	37 (59.7)	25 (40.3)	1		1	
**Marital Status**						
Single	7 (30.4)	16 (69.6)	1.09 (0.48-2.87 )	0.94		
Married	73 (32.3)	153 (67.7)	1			
Widowed	20 (60.6)	13 (39.4)	0.31 (0.14-0.87)	0.08		
Divorce	16 (40)	24 (60)	0.71 (0.32-1.86)	0.52		
**Religion**						
Orthodox	53 (35.3)	97 (64.7)	1			
Muslim	10 (55.6)	8 (44.4)	0.44 (0.16-1.17)	0.1		
Protestant	45 (34.6)	85 (65.4)	1.03 (0.63-1.69)	0.9		
Hawaryat	8 (33.3)	16 (66.7)	1.09 (0.44-2.7)	0.85		
**Income in ETB**						
<2000	55 (73.3)	20 (26.7)	1			
2000-5000	57 (26.0)	162 (74.0)	7.8 (4.3-14.16)	0.000	4.51 (2.18-9.34)	<0.001
>5000	4 (14.3)	24 (85.7)	16.5 (5.09-53.5)	0.000	12.45 (3.2-47.77)	<0.001
**Educational Status**						
Illitrate	17 (65.4)	9 (34.6)	1			
Read and write only	29 (59.2)	20 (40.8)	1.3 (0.49-3.5)	0.6		
Primary	21 (38.2)	34 (61.8)	3.06 (1.16-8.1)	0.024		
Secondary	22 (33.8)	43 (6.2)	3.69 (1.4-9.62)	0.008		
Teritary	27 (21.3)	100 (78.7)	6.99 (2.8-17.4)	0.001		
**Occupation**						
Unemployed	22 (73.3)	8 (26.7)	1		1	
Daily	28 (71.8)	11 (28.2)	1.08 (0.37-3.14)	0.89	0.84 (0.25-2.75)	0.77
Merchant	27 (41.5)	38 (58.5)	3.87 (1.5-9.99)	0.005	2.45 (0.82-7.27)	0.106
Governent	25 (14.9)	143 (85.1)	15.7 (6.3-39.2)	0.000	7.58 (2.7-21.23)	<0.001
Other	14 (70.0)	6 (30.0)	1.18 (0.34-4.13)	0.797	0.79 (0.19-3.23)	0.74
**Ethnicity**						
Bench	60 (37.3)	101 (62.7)	1			
Keffa	19 (26.8)	52 (73.2)	1.63 (0.88-3)	0.12		
Amhara	10 (31.2)	22 (68.8)	1.3 (0.58-2.95)	0.52		
Oromo	11 (61.1)	7 (38.9)	0.38 (0.14-1.03)	0.06		
Sheko	16 (40)	24 (60)	0.89 (0.44-1.81)	0.75		
**Any relative who have prostate problem**						
Yes	3 (15)	17 (85)	3.39 (0.97-11.88)	0.06		
No	113 (37.4)	189 (62.6)	1			
**Drinking alcohol**						
Yes	14 (46.7)	16 (53.3)	1			
No	102 (34.9)	190 (65.1)	1.63 (0.77-3.5)	0.21		
**Smoke**						
Yes	18 (36.7)	31 (63.3)	1			
No	98 (35.9)	175 (64.1)	1.04 (0.55-1.95)	0.91		

Regarding age, respondents who are 40–55 years old had a 6.16 times higher level of prostate cancer awareness than those whose age is >65 (AOR = 6.16).

Concerning occupation, those who were government employees had a 4.67 times higher level of prostate cancer awareness than those unemployed (AOR = 4.67). Regarding economic status, those whose monthly income level is >5,000 birr had a 12.45 times higher level of prostate cancer awareness compared to those whose income is <2,000 birr

## Discussion

The findings of the study showed that 64% of men residing in Mizan Aman town have a high level of prostate cancer awareness. However, the level of awareness regarding prostate cancer varies by sample characteristics such as age, economic status, and occupation. This may indicate that there are a significant number of men who might have a late diagnosis of prostate cancer, which could lead to a poor prognosis. The percentage level of prostate cancer awareness in the current study is far lower as compared with that found in a study conducted in Oyo State, Nigeria, which was 80% (23). This variation can be attributed to differences in sample characteristics and information about prostate cancer. In Nigeria, more research was done on the awareness, perception, knowledge, attitude, and practice of prostate cancer. This may indicate more cases of undiagnosed prostate cancer in Ethiopia than in Nigeria.

However, according to a case study done in rural Mhondoro-Ngezi, Kadoma District, Zimbabwe, the percentage of prostate cancer awareness is 21%. This might be attributed to the differences in the sociodemographic status of the study participants due to the rural residency and study design that the study used (24).

Other important findings of the current study are that age, economic status, and occupation are significantly associated with the level of awareness. Relatively younger age, higher income, and being government-employed increase the chance of being aware of prostate cancer. For example, those with poor awareness of prostate cancer were older compared to those with good awareness.

Regarding occupation, those who were government employees are almost five times more likely to suffer from prostate cancer than those who were unemployed. This is in line with the study findings on adult males over the age of 40 years in Benin City, Nigeria (13). This shows that civil servants were associated with prostate cancer awareness and that government employees were more knowledgeable about prostate cancer than others. This could be due to they had to get more information than others.

### Conclusion and recommendation

This study has revealed that more than half of men residing in Mizan Aman town had a high level of prostate cancer awareness. First classification age, better economic status, and employment increased the likelihood of prostate cancer awareness. A collective effort from different stakeholders should be geared toward designing an awareness creation strategy using the input of the current study. In collaboration with the Mizan Aman Education Bureau, the Mizan Aman Health Bureau should develop adult-centered educational programs created specifically for those with low economic status and those unemployed and older. Furthermore, a large-scale, multicentered, and mixed-methods research is required to be conducted in the future.

## Data availability statement

The datasets presented in this study can be found in online repositories. The names of the repository/repositories and accession number(s) can be found in the article/Supplementary Material.

## Ethics statement

The studies involving human participants were reviewed and approved by Jimma University Ethical review board. The patients/participants provided their written informed consent to participate in this study.

## Author contributions

AA conceived the study, drafted the proposal, analyzed the collected data, and prepared the final manuscript for publication. MA was involved in the design of the study and in revising the manuscript critically for important intellectual content. All authors read and approved the final manuscript.

## Funding

The authors received funding for this study from Jimma University.

## Conflict of interest

The authors declare that the research was conducted in the absence of any commercial or financial relationships that could be construed as a potential conflict of interest.

## Publisher’s note

All claims expressed in this article are solely those of the authors and do not necessarily represent those of their affiliated organizations, or those of the publisher, the editors and the reviewers. Any product that may be evaluated in this article, or claim that may be made by its manufacturer, is not guaranteed or endorsed by the publisher.

## Abbreviations

DRE, digital rectal examination; NCD, non-communicable disease; NHIS, National Health Insurance Scheme; PI, principal investigator; PSA, prostate-specific antigen; WHO, World Health Organization.
